# The Nociceptive and Anti-Inflammatory Effects of *Artemisia dracunculus* L. Aqueous Extract on Fructose Fed Male Rats

**DOI:** 10.1155/2015/895417

**Published:** 2015-06-11

**Authors:** Shahraki Mohammad Reza, Mirshekari Hamideh, Samadi Zahra

**Affiliations:** ^1^Department of Physiology, Faculty of Medicine, Zahedan University of Medical Sciences and Health Services, Zahedan, Iran; ^2^Zahedan Health Center, Zahedan University of Medical Sciences, Zahedan, Iran; ^3^Zahedan University of Medical Sciences and Health Services, Zahedan, Iran

## Abstract

*Aim & Objective*. *Artemisia dracunculus* L. (*Tarragon*) species have been used as a traditional medicine. The present study was designed to evaluate the nociceptive and anti-inflammatory effects of *A. dracunculus* L. leaf aqueous extract on fructose drinking water (FDW) in male rats. *Materials & Methods*. Forty-eight Wistar-albino male rats weighing 200–250 g were divided into control (C), control extract (CE), FDW, and FDWE groups (*n* = 12). Group C did not receive any agents; Group CE did 100 mg/kg *A. dracunculus* L. aqueous extract on a daily basis for duration of four weeks. FDW Group received fructose drinking water (10%, weight/volume) but did not receive any agents during trial period. FDWE group received 100 mg/kg *A. dracunculus* L. aqueous extract during trial period. At the end of experiment, a biphasic pain response was induced following interplanetary injection of formalin (50 *µ*L, 1%). Obtained data were analyzed using SPSS software version 17 and using ANOVA and Tukey post hoc tests. Results were expressed as mean ± SE. Statistical differences were considered significant at *P* < 0.05. *Results*. Our findings revealed that acute and chronic pain scores in FDW group are significantly higher than other ones and *A. dracunculus* L. aqueous extract causes significant decreasing of this parameter in FDWE group (*P* < 0.001). Moreover, IL6 and TNF values in this group were significantly decreased compared to FDW group (*P* < 0.05). *Conclusion*. Results in the present study show that FDW causes the pain response score to increase and cause proinflammatory cytokines in rat model but *A. dracunculus* L. leaf aqueous extract improves values of these parameters.

## 1. Introduction

Metabolic syndrome (Met S) is illustrated as clustering several metabolic abnormalities of an individual based on hyperlipidemia, abdominal obesity, and insulin resistance [1]. This disorder is associated with low-grade chronic inflammatory activity state and it increases the cytokines serum concentration [2]. In addition, there are associations among insulin resistance, abdominal obesity, and serum immune markers such as IL6 and TNF*α* [3]. Moreover, metabolic syndrome related to insulin resistance (IR) is a state in that insulin is higher than normal concentration and it plays an important role in the pathophysiology of most common human diseases such as type 2 diabetes mellitus, hypertension, and coronary heart disease [4]. A mixture of several botanical medicines is used to treat diabetes mellitus and insulin resistance accompanied by hypertension in fructose-fed rats (FFR) [5].* Artemisia* is a widespread traditional plant and it has varied genus with different species of the family* Asteraceae* as well as great therapeutic and economic importance [6]. A botanical extract obtained from* Artemisia dracunculus (A.D.L)* has been shown to improve insulin sensitivity by increasing cellular insulin signaling in vitro in muscle cell culture [7]. Moreover, investigation confirmed that Artemisinin, a component taken from* Artemisia annual*, can affect as a potential contraceptive agent with antimalarial activity [8]. The essential oil obtained from the aerial parts of* AD* has orally been used as an antiepileptic remedy in traditional medicine [9]. Additionally, Artemisinin is an intrinsic product showing powerful anticancer activity in different types of human tumors [10]. Furthermore, experiments indicated that Artemisinin induced the generation of regulatory T cells with extraordinarily inhibitory effect on IL-17 production, diminishing the level of IL-6 in mouse model [11]. Based on above investigations, the aim of the present study was to evaluate the nociceptive and anti-inflammatory effects of* Artemisia dracunculus (AD)* leaf aqueous extract on FDW male rats.

## 2. Materials & Methods

Forty-eight Wistar-albino adult male rats weighing 200–250 g were selected from Medical Sciences of Zahedan University animal house and they were reserved in individual cages (one rat in each cage). The rats had free access to water and food and they were kept in a room at 23 ± 2°C with a fixed 12:12-h artificial light/dark period (timer model: SUL180a, AC220V, China, 6 Am to 6 Pm) and a suitable humidity of 45–60%. After a week of accommodation, the rats were randomly divided into C, CE, FDW, and FDWE groups (*n* = 12 in each group) as follows.

 Group C did not receive any agents during experiment period.
 Group CE received daily water tap, rodent's diet, and 100 mg/kg* AD* aqueous extract for four weeks by gavages.
 Group FDW received fructose-enriched water (10% w/v) and rodent's diets during experimental period but group FDWE did intake fructose-enriched water (10% w/v) daily, rodent's diets, and 100 mg/kg* ADL* aqueous extract at this time by gavages.

### 2.1. Preparation of the Extracts

Plants of the genus* Artemisia* (family Asteraceae) were collected from local area around Tehran, the capital of Iran, in August 2013 and identified by the Biology Taxonomy Centre in Science Faculty of Sistan and Baluchestan University, Zahedan, Iran.* Artemisia dracunculus* aerial parts were separated, shade-dried in a room temperature, and then converted into powder by hands. Extraction was performed by mixing 20 grams of powder in 200 mL of distilled water for 24 hours in soxhlet extractor [12]. The prepared extract was filtered through a gauze cloth followed by filtration through a normal filter paper Whatman no. 1 [12]. The product was a dark brown aqueous extract dried afterward in incubator for one day at 45° and sustained in appropriate temperature (regenerator).

### 2.2. Pain Behavioral Response Scoring

Acute pain was assessed using the 1% formalin test [13]. The rats were located in open Plexiglas observation chambers for 30 minutes to permit them to provide accommodation to their condition. Then they were removed for formalin administration. After formalin administration, each rat was placed in a Plexiglas observation box measuring 40 cm × 20 cm × 20 cm. A mirror was placed under chamber floor at a 45° angle to allow a clear view of the rat's paws. Pain behavior responses were recorded beginning from the subcutaneous injection of 1% formalin (50 *μ*L/paw) with a 30-gauge needle into the dorsal surface of the right hind paw. Formalin induced biphasic flinching behavior in rats.

The primary acute phase (0–10 min) was then followed by a quite short period, which was followed by an expanded constant response (15–60 min). Pain behavioral responses were measured every minute and continued at 5-minute intervals for one hour [13]. The scores follow the Dubinson method:

 1 = the injected paw which is not superior indicates no pain;
 2 = the injected paw with little or no weight on it, with no toe splaying, indicates mild pain;
 3 = the injected paw elevated and the heel is not in contact with any surface indicates moderate pain;
 4 = the injected paw licked, bitten or taken aback indicates severe pain.

At the end, animals were deeply anesthetized by diethyl ether (Merck Germany) and sacrificed and blood samples were immediately collected from cervical vessels. All blood samples were collected in ordinary vials and centrifuged at 3000 rpm for 10 minutes in order to separate serum. Serum was removed (BH-1200 type Iran) and stored at −70°C for further analyses. Serums IL6 and TNF were measured by sensitive rat kit (Cusabio Biotech Co. Ltd., China), using double antibody enzyme-linked immunosorbent assay (ELISA). These experiments on animals were carried out in accordance with recommendations from the pronouncement of Helsinki and internationally conventional principles for the use of experimental animals, and they received institutional ethical approval from the committee for Animal Research of Zahedan University of Medical Sciences.

The normal distribution of data was approved by Kolmogorov-Smirnov test, and then all data were analyzed by SPSS software version 17, via ANOVA and Tukey post hoc test. The results were expressed as mean ± SE. Statistical differences were considered significant at *P* < 0.05.

## 3. Results

**Figure 1:**
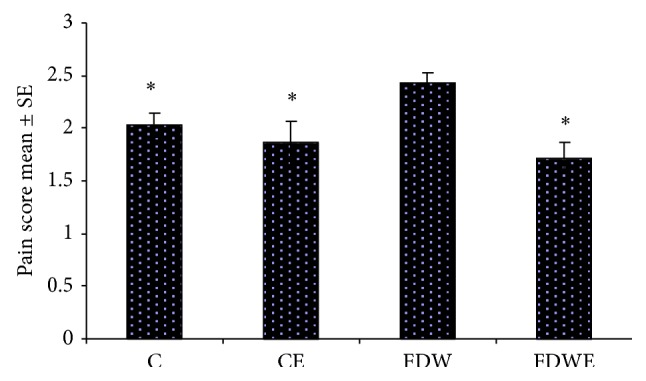
Mean acute pain score in C, CE, FDW, and FDWE. *n* = 12, mean ± SE and ^*^ =  *P* < 0.05. Based on ANOVA and Tukey post hoc, consumption of fructose dirking water enhances the acute pain response in rats but* Artemisia* aqueous extract administration results in decreasing of the acute pain response in FDWE group.

**Figure 2:**
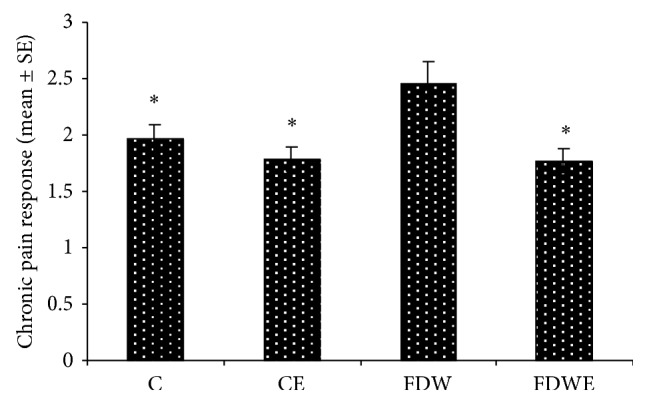
Chronic pain in C, CE, FDW, and FDWE. *n* = 12, ^*^+ *P* < 0.05, mean ± SE. Based on statistical tests ANOVA and Tukey post hoc, consumption of fructose drinking water enhanced the chronic pain response in rats but* Artemisia* aqueous extract administration leads to decreasing of this parameter in FDWE group.

**Figure 3:**
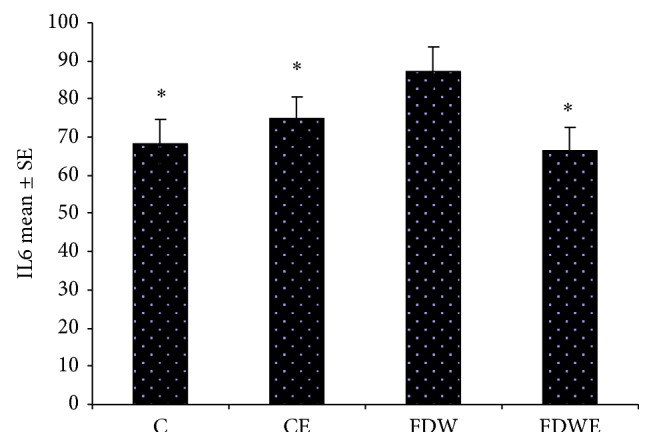
IL6 (ng/L) value in C, CE, FDW, and FDWE groups. ^*^ =  *P* < 0.05, *n* = 12. Based on statistical tests ANOVA and Tukey post hoc, consumption of fructose dirking water enhanced the cytokines value (IL6) in rats but* Artemisia* aqueous extract administration causes this value to decrease for FDWE group.

**Figure 4:**
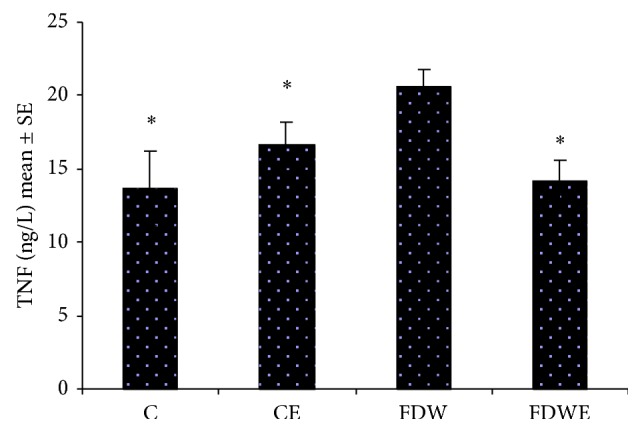
Tumor necrosis factor (ng/L) in C, CE, FDW, and FDWE. ^*^ =  *P* < 0.05, mean ± SE, *n* = 12. Based on statistical tests ANOVA and Tukey post hoc, consumption of fructose drinking water improved the TNF value in male rats. On the other hand,* Artemisia* aqueous extract administration causes this parameter to fall in FDWE group.

Results obtained from the present study indicated that insulin-resistance indexes (IRI) in FDW group (0.14) were significantly higher than those of C and CE groups (0.07, 0.12). In addition, IL6 and TNF value in FDW groups increased significantly in comparison with C and CE groups. Moreover, IRI (0.12), IL6, and TNF values in FDWE group decreased noticeably compared to those of FDW group (Figures 3 and 4). On the other hand, acute and chronic pain response scores in FDW extract group fell considerably down comparison with FDW group (Figures 1 and 2).

## 4. Discussion

Fructose is a monosaccharide found in fruits and used as sweetener in foods and drinks, and its consumption is related to incidence of abdominal obesity, insulin resistance, and type II diabetes mellitus [14]. Insulin and leptin are two important hormones interfering with the regulation of food intake and body weight gain in all vertebrata and human [15]. However, dietary fructose contributes to increased energy input and weight gain and alters blood lipid and carbohydrate homeostasis [15]. Insulin resistance and hyperinsulinemia are common results among patients with MS, insulin resistance, type II diabetes, and essential hypertension, which are high risk factors of cardiovascular diseases and have an important role in the development of coronary artery disease [5]. Our finding in this study revealed that FDW consumption, in a month, induced insulin resistance index (IRI), hyperinsulinemia a serum proinflammatory cytokines values such as IL6 and TNF in male rats. These results were confirmed by previous studies indicating that fructose-fed and FDW cause central obesity, insulin resistance, and hyperinsulinemia to increase in rats [4]. Previous studies indicated that TNF serum concentration elevated and altered insulin signaling in skeletal muscles cell membranes in insulin resistance disorders (type II diabetes and MS), but* Artemisia dracunculus* L. extract improved insulin action by increasing insulin signaling in skeletal muscle [16]. Investigational evidence determined that metabolic pathways associated with glucose transport, glycolysis, and cell signaling is probably influenced by* Artemisia dracunculus* L. aqueous extract and modulates carbohydrate metabolism as well as translocation of GLUT4 to the plasma membrane skeletal cells [17]. This observation pointed out that a molecular mechanism may be influenced by the* Artemisia dracunculus* L. aqueous extract components and it enhances glucose uptake, glucose transport in that sensitive-to-insulin cells, improving whole body insulin sensitivity [17]. Our results in this study showed that consumption of* Artemisia* aqueous extract causes IRI to improve in FDWE group compared to that of FDW group and it is in agreement with those of literatures. Some experimentation has shown that diabetic and insulin-resistant patients have higher adipose tissues-derivatives TNF*α* in comparison with normal samples [18]. On the other hand, TNF inhibits insulin-stimulated activation of the tyrosine kinas in rat adipocytes and finally suppresses glucose transporter 4 (GLUT4) and glucose intake in these cells [19].* Artemisia* crude extract has shown anti-inflammatory effects, but its associated anti-inflammatory mechanisms are not clear [20]. Fructose-drinking water treated by* Artemisia* aqueous extract had significant enhancement to insulin-responsive [21]. Our results expressed that TNF serum concentration in FDW rats is significantly higher than control and it is confirmed by previous studies. Moreover,* Artemisia dracunculus* L. aqueous extract improves these parameter values in FDWE group. Previous studies indicated that* Artemisia* hydromethanolic extract has shown an antinociceptive property in normal mice [22]. In addition, experimental studies revealed that* Artemisia* species has antihyperalgesic and antiallodynic properties through the inhibition of different seditious pains signaling a significant component for the treatment of inflammatory pain [23]. The results in the present study indicated that* Artemisia* aqueous extract decreased the acute and chronic pain responses in FDWE group compared to FDW group. Maham et al. in 2014 confirmed that essential oil of* Artemisia dracunculus* (EOAD) has peripheral and central antinociceptive effects in normal mice and it seems that a variety of mechanisms compared to opioid receptors are involved in the analgesic effect of EOAD [24]. Our results in this study showed that acute and chronic pain score in FDWE group is significantly lower than that of FDW group and it is confirmed by prior studies. Literature investigated that* Artemisia* species have shown a high ferric reducing antioxidant properties against oxidant damages [25]. In addition Saoudi et al. in 2010 reported that* Artemisia campestris* leaves aqueous extract contains a lot of cations such as K(+), Na(+), and Ca(++) and antioxidant such as polyphenols and scavenging activities and superoxide anion which has antioxidant and protective effects against liver oxidant [26]. Our finding in the present study showed that* Artemisia dracunculus* L. leaves aqueous extract have anti-inflammatory and analgesic effects and supported by literatures.

## 5. Conclusion

Our finding in the current study indicated that* Artemisia dracunculus* leaves aqueous extract has antinociceptive and anti-inflammatory effects in FDW rats.
